# Acute physiological, perceived exertion and enjoyment responses during a 4-week basketball training: a small-sided game vs. high-intensity interval training

**DOI:** 10.3389/fpsyg.2023.1181646

**Published:** 2023-06-26

**Authors:** Jinshu Zeng, Haris Pojskic, Jing Xu, Yuanhong Xu, Fei Xu

**Affiliations:** ^1^School of Physical Education, Hangzhou Normal University, Hangzhou, China; ^2^Department of Sports Science, Faculty of Social Sciences, Linnaeus University, Kalmar, Sweden

**Keywords:** internal load, training evaluation, exercise adherence, training periodization, women players, self-determination theory

## Abstract

**Introduction:**

Although previous research found that small-sided game (SSG) training was more enjoyable than high-intensity interval training (HIT) in various sports, no data were provided during longer training period in basketball. Furthermore, the comparison of internal loads between the two training approaches needs to be further examined. Thus, this study aimed to examine the acute physiological, perceived exertion and enjoyment responses during 4-week progressive basketball SSG or HIT programs.

**Methods:**

Nineteen female collegiate basketball players were randomly assigned to two groups that performed either HIT (*n* = 10) or SSG (*n* = 9) 3 times per week for 4 continuous weeks. Average and percentage of maximal heart rate (HR_mean_ and %HR_max_), rating of perceived exertion (RPE), and physical activity enjoyment (PACES) were determined during each training session.

**Results:**

There was a main group effect in PACES (*p* < 0.001; $\eta_{p}^{2}$ = 0.44, moderate), and SSG had higher PACES than HIT in each week (*p* < 0.05). There were no significant interactions or main group effects in HR_mean_, %HR_max_ or RPE, but a main time effect was found in HR_mean_ (*p* = 0.004; $\eta_{p}^{2}$ = 0.16, minimum), %HR_max_ (*p* < 0.001; $\eta_{p}^{2}$ = 0.25, minimum), and RPE (*p* < 0.001; $\eta_{p}^{2}$ = 0.31, moderate), respectively. In the SSG group, although no significant differences were found in HR responses, %HR_max_ was below 90% in week 1 and week 2. Accompanied with changes in %HR_max_, RPE in week 1 and week 2 was lower than that in week 3 and week 4 (*p* < 0.05).

**Conclusion:**

Our findings suggest that SSG and HIT elicit similar acute HR response and RPE level, but SSG is perceived as more enjoyable and therefore it is more likely to increase exercise motivation and adherence comparing to HIT. Moreover, it seems that half-court, 2 vs. 2 SS Gtraining format with modified rules and lasting ≥ 7.5 min should be prescribed as an enjoyable training alternative to provide optimal cardiovascular stimuli (> 90% of HR_max_) for female basketball players.

## 1. Introduction

Basketball is an intermittent, court-based team sport characterized by high aerobic and anaerobic demands, frequent changes of movement and various technical-tactical scenarios (Stojanovic et al., 2018). Data from game activities indicate the predominant utilization of aerobic metabolism (Ben Abdelkrim et al., 2010; Latzel et al., 2018). Aerobic capacity contributes to the repetition of high-intensity movements, as well as the ability to maintain high quality of these movements with limited recovery time (Ben Abdelkrim et al., 2010; Dupont et al., 2010).

For effectively improving aerobic capacity, training at high heart rate (HR) zones (above 90% of HR_max_) is considered to be more effective than lower HR zones training (Delextrat and Kraiem, 2013; Malone et al., 2019). In this regard, high-intensity interval training (HIT) and small-sided game (SSG) have been widely used to improve aerobic fitness in basketball players because these training approaches can elicit cardiovascular response above 90% of HR_max_ (Delextrat and Martinez, 2014; Zeng et al., 2022). SSG includes a series of shorter-duration games with smaller numbers of players and modified rules compared to real matches (Clemente, 2016), and HIT includes a series of brief intermittent workouts (e.g., sprints) performed at a maximum or near-maximal effort (Gibala and McGee, 2008). Moreover, to achieve optimal effects during a training period lasting several weeks or months, training load (i.e., durations, frequencies and intensities) should be gradually increased in response to advanced training-induced adaptations (Smith, 2003; Triplett and Chandler, 2017). With this in mind, progressive SSG and HIT training programs are frequently applied and compared in previous studies (Delextrat and Martinez, 2014; Zeng et al., 2022). For instance (Delextrat and Martinez, 2014) showed that both SSG and HIT interventions performed twice per week for 6 weeks significantly improved aerobic capacity in junior male basketball players. Moreover, a shorter SSG and HIT training period (i.e., 4 weeks) with a higher training frequency (i.e., 3 times per week) showed to be effective in enhancing aerobic capacity in female basketball players (Zeng et al., 2022).

However, although the aforementioned studies evaluated outcomes of interventions (pre- vs. post-intervention), a lack of data detailing physiological and psychological responses limits the ability to comprehensively assess program implementation (Moore et al., 2015). Monitoring the precise physiological (e.g., HR, RPE, blood lactate) and psychological (i.e., enjoyment) responses during training sessions enables practitioners and researchers to understand the internal load imposed on players and optimize and adjust designed training programs when needed (Reina et al., 2020; Twist et al., 2023). This is particularly important for SSG intervention given its predominant use in basketball.

To date, numerous studies have focused on determining the influence of team sizes, court size, game rules, and work: rest time on the physiological demands and perceived exertion encountered by players during SSG (Clemente, 2016; O'Grady et al., 2020). The findings from the majority of studies suggest that SSG with smaller team sizes (e.g., 2 vs. 2, 3 vs. 3; Klusemann et al., 2012; Conte et al., 2016), bigger playing areas (Atli et al., 2013) and longer duration bouts (Klusemann et al., 2012) can evoke higher HR and self-perceived exertion (RPE) responses. Moreover, the game rules of time constraints (Camacho et al., 2020) and dribbling prohibitions (Conte et al., 2015) were utilized to increase HR responses. However, few studies have compared the impact of different training approaches (e.g., SSG vs. HIT) on exercise intensity in male/female basketball players, showing that SSG and HIT elicited similar HR responses (Delextrat and Martinez, 2014). Nonetheless, inconsistent results in RPE of the two training approaches remain in existing studies, with RPE of HIT higher than or similar to that of SSG (Delextrat et al., 2018; Arslan et al., 2020). Thus, more studies are needed to determine the physiological and perceived exertion responses during SSG and HIT training.

On the other hand, psychological responses during and following exercise are seen as key factors for predicting future exercise intentions, behavior, and adherence (Stork et al., 2018). Specifically, the enjoyment towards an exercise, as usually determined by physical activity enjoyment scale (PACES), is an important predictor of exercise motivation and adherence (Selmi et al., 2020), both of which can lead to players continuing to play at the professional stage. Previous research found that SSG were more enjoyable (higher PACES) than HIT in soccer players (Los Arcos et al., 2015; Arslan et al., 2020). Also, it was observed that SSG provided higher enjoyment than HIT in young tennis players (Kilit and Arslan, 2019). Although these studies (Kilit and Arslan, 2019; Arslan et al., 2020) provided useful insight into enjoyment responses during SSG and HIT, no data were provided across all sessions per week during a longer training period (e.g., 4 weeks). In addition, there is a lack of data detailing players’ enjoyment responses in female basketball players, thus calling for further investigation in this area.

Therefore, we aimed to examine the acute physiological, perceived exertion and enjoyment responses of female basketball players in 4-week SSG or HIT programs. It was hypothesized that SSG and HIT would elicit similar physiological and perceived exertion responses, while SSG would have higher enjoyment responses than HIT in each training session. In addition, the hypothesis was based on the self-determination theory (SDT; Deci and Ryan, 2000) that provides theoretical framework for understanding potential differences in induced psychological outcomes (i.e., perceived exertion and enjoyment) between SSG and HIT. In brief, it is known that stimulating environmental factors can facilitate the satisfaction of the three basic psychological needs (i.e., autonomy, competence and relatedness) and when they are met they increase self-determined (autonomous), intrinsic motivation, which in turn positively affects perceived effort and enjoyment response (Deci and Ryan, 2000; Sarrazin et al., 2002; Pope and Wilson, 2012; Sheldon et al., 2013; Monteiro et al., 2018; Lourenço et al., 2022).

## 2. Materials and methods

### 2.1. Participants

This study was based on a randomized parallel matched-group design. Generally, 24 female collegiate basketball players were recruited from one basketball team competing in a regional league. Players were matched based on their playing positions (center, forward, and guard) and training years, and then randomly assigned to a HIT group (*n* = 12) or a SSG group (*n* = 12). The inclusion criteria included regular participation in training sessions and tournaments, and no lower limb injury and/or surgery happened in the past 6 months. The exclusion criteria for players’ data analysis included more than twice missing training (*n* = 3) or occurring lower limb injuries (*n* = 2). Thus, the final sample included 19 players (SSG: *n* = 9, age 20.0 ± 1.3 years, height 166.1 ± 6.6 cm, weight 59.2 ± 9.2 kg, maximal HR [HR_max_] 198 ± 6.1 b min^−1^, maximal oxygen uptake [VO_2_ max] 44.4 ± 1.2 mL/kg/min, training experience 5.8 ± 2.0 years; HIT: n = 10, age 19.8 ± 0.8 years, height 165.1 ± 5.5 cm, weight 56.6 ± 11.0 kg, maximal HR [HR_max_] 198.9 ± 4.8 b∙min^−1^, maximal oxygen uptake [VO_2_ max] 43.8 ± 1.4 mL/kg/min, training experience 5.6 ± 1.8 years). Verbal inquiries by the coaching staff revealed that players’ menstrual cycles were stable in the past 3 months, and they normally participated in training and competitions during menstrual cycles. All players were informed about the experimental procedures, potential benefits, and risks before providing written consent to participate. Players were made aware that they could withdraw from the study at any time without penalty. The study was approved by the ethical standards of the local ethical committee (Number: IR00350-SPT-2020) and followed to the recommendations of the Declaration of Helsinki.

### 2.2. Variables

One week before the intervention period, body height (BH), body mass (BM), maximum heart rate (HR_max_) and maximum running velocity (V_IFT_) during the 30–15 intermittent fitness test (30-15_IFT_) were assessed. BH was measured to the nearest 0.1 cm with a portable stadiometer (Seca, mod206 Birmingham United Kingdom) and BM to the nearest 0.1 kg with a portable scale (Seca, mod762, Birmingham United Kingdom). Players’ HR was measured during training, while their perceived exertion (RPE) and enjoyment responses were measured after training.

Players’ HR_max_, V_IFT_ and aerobic fitness were estimated using the 30-15_IFT_ (Buchheit, 2010). The test has been shown to have high reliability (ICC = 0.96; Buchheit, 2010). It was performed on a full basketball court (28 × 15 m) and consisted of 30-s shuttle run separated by 15-s passive recovery. The initial velocity was set at 8 km/h and increased by 0.5 km/h every 45 s thereafter. The test was terminated when players were unable to sustain the requisite running speed. HR at that time was recognized as HR_max_, while the velocity obtained in the final fully completed stage was taken as V_IFT_. The HR responses were assessed using Polar V800 (Polar Electro Oy, Finland) and then exported and analyzed using Kubios HRV Standard 3.4.1 (University of Eastern Finland, Kuopio, Finland). To estimate mean heart rate (HR_mean_, beats/min) and the percentage of maximal HR (%HR_max_) reached in each SSG and HIT session, players’ HR responses were continuously monitored (Reina et al., 2018, 2020).

Players’ RPE was assessed using the Borg CR-10 category-ratio scale, which ranges from “very light activity” (1) to “max effort activity” (10; Borg, 1998). Players were required to verbally express their RPE immediately after each SSG and HIT session. Players had used the CR-10 for 4 weeks prior to the study to evaluate their regular exercise intensities.

Players’ enjoyment responses to training were determined using a short–term PACES (Graves et al., 2010). Players filled out the PACES anonymously 3 min after each SSG and HIT session. The short-term PACES includes 5 items scored on a 1–7 Likert scales, and the total enjoyment responses for each training and for each player were summed to yield a score ranging from 5 to 35 (Graves et al., 2010). Players filled out the PACES anonymously to ensure the accuracy of perceived enjoyment. The PACES has been found to have high reliability and validity in physical activity environments (Kendzierski and DeCarlo, 1991).

### 2.3. Training programs

**Figure 1:**
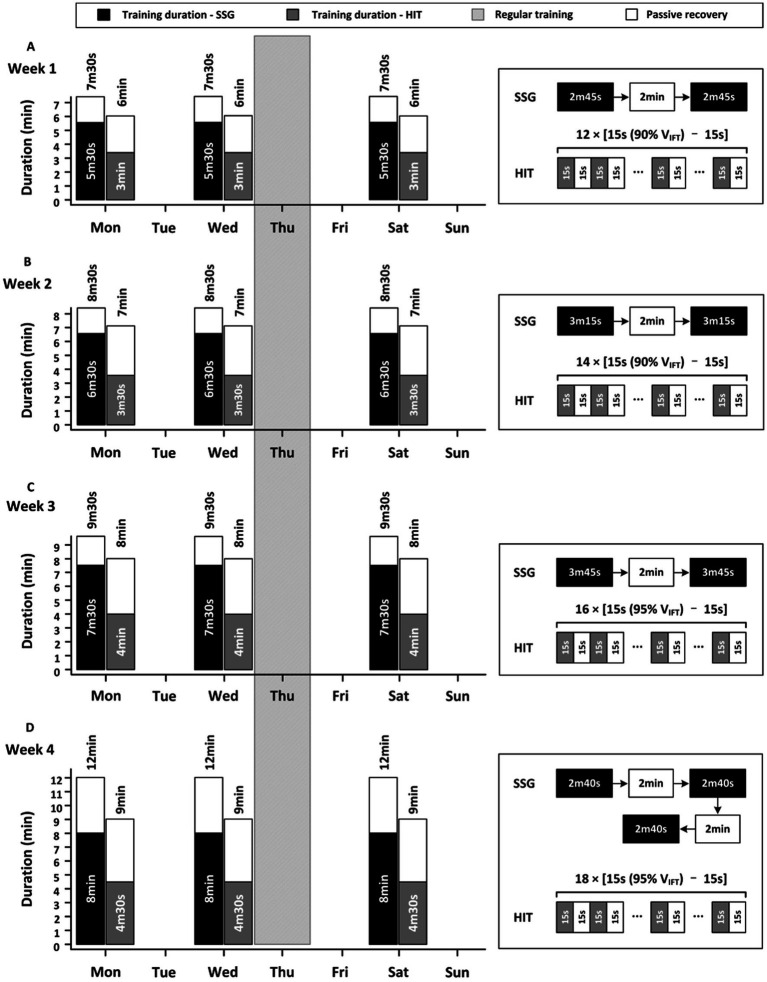
The training duration and regimen of each SSG and HIT session (Monday, Wednesday, and Saturday) in week 1 **(A)**, week 2 **(B)**, week 3 **(C)**, and week 4 **(D)**. Only regular training program performed in Thursday; 12 × [15 s (90%V_IFT_) – 15 s] means 12 bouts of 15-s high-intensity running at a speed equal to 90% of the speed achieved in the final completed stage of the 30–15 intermittent fitness test, followed by 15-s passive recovery.

The 4-week training program was conducted during the pre-season. A typical week during this period consists of four 2-h training sessions that include various intensities of running, core conditioning, specific technical and tactical drills, and matches. SSG or HIT were performed 3 times per week in addition to regular practice sessions. Both interventions followed a progressive overload plan involving gradual increase in training stress (e.g., exercise duration, number of bouts and repetitions) over time (Figure 1). The design of the training matched duration in SSG and HIT was based on the recommendations of previous studies (Buchheit et al., 2009; Delextrat and Martinez, 2014). SSG and HIT were always performed at the beginning of each training session after a 15-min standardized warm-up that includes low-intensity running, dynamic stretching, and ball practice (dribbling, shooting, and layup). Prior to the training interventions, players have trained for 4 weeks to prepare their bodies for the intense exercise.

The SSG sessions involved 2 players per team (2 vs. 2) on half of the basketball court (15 × 14 m). Drills were played like a competition continuously for 2 min45 s–3 min45 s, followed by 2-min passive recovery between bouts. Scores were kept during games and strong verbal encouragements were provided by the coaching staff to improve players’ motivation. Each SSG was refereed by assistant coaches who were qualified to referee. The following SSG rules were adopted: (a) only man-to-man defense to standardize technical–tactical parameters (Conte et al., 2015); (b) no free-throws or time-outs (Delextrat and Kraiem, 2013); (c) the shot clock was set to only 12 s (Klusemann et al., 2012); (d) if an offensive rebound was obtained, the players could continue to attack the basket directly; (e) if a defensive rebound was obtained or points scored, the players had to take the ball to the center circle before attempting to score; (f) after a change of possession (e.g., steal, defensive rebound) or points scored, players were allowed to steal the ball from the team attempting to bring the ball to the center circle; (g) in the event of fouls, turnover or ball out-of-bounds, the game was restarted when an offensive player ran to the nearer sideline and caught the spare ball from an assistant (Conte et al., 2016). During each SSG session, players were randomly assigned a pairing (consisting of a guard, and either a forward or a center), and new pairings were formed in the following session.

The HIT sessions included a series of intermittent running at 90–95% of players’ V_IFT_ for 15 s on a 20-m-long field integrating 180° changes of direction, followed by 15-s passive recovery. During each 15-s running, players should start from their own position (based on their target running distance) and finish all together on the same line. During the 15-s recovery period, players should walk to their starting line and wait for the next 15-s running.

### 2.4. Statistical analysis

Data analyses were performed using the open-source statistical software JASP.^1^ The normality of all data was checked using the Shapiro–Wilk test. The homogeneity of variance was confirmed with a Levene test. Mixed two-way ANOVAs with one “between” factor (group: SSG and HIT) and one “within” factor (time: week 1, 2, 3 and 4) was used to determine changes in physiological, perceived exertion and enjoyment responses in both interventions. Significant effects were subsequently examined using the Bonferroni post-hoc test. Partial eta squared ( $\eta_{p}^{2}$ ) was calculated to estimate main effects and interaction effects. The thresholds for $\eta_{p}^{2}$ were as follows: < 0.04, no effect; 0.04–0.25, minimum effect; 0.25–0.64, moderate effect; > 0.64, strong effect (Ferguson, 2016). Hedges’ *g* was used to indicate the effect size for pairwise comparisons and interpreted as followed: < 0.2, trivial; 0.2–0.6, small; 0.6–1.2, moderate; 1.2–2.0, large; > 2.0 very large (Hopkins et al., 2009). The level of significance was set at *p* < 0.05. Data were presented as mean and standard deviation (*M* ± *SD*) or mean difference (*MD*) and 95% confidence intervals (95% *CI*).

## 3. Results

### 3.1. Physiological responses

**Figure 2:**
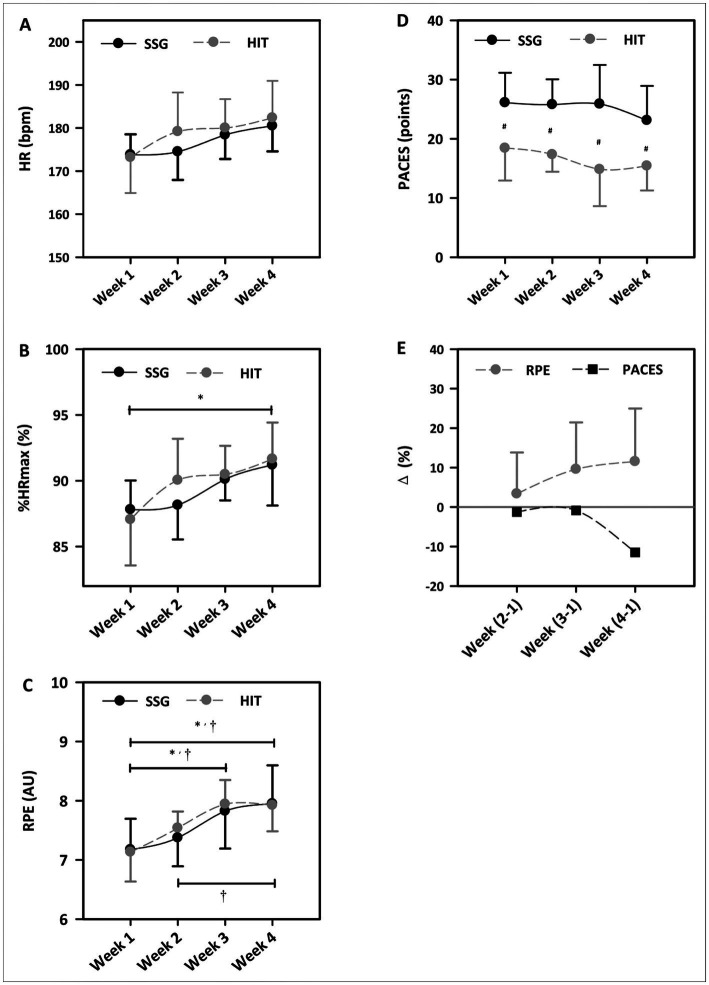
HR_mean_ **(A)**, %HR_max_ **(B)**, RPE **(C)**, and PACES **(D)** recorded in the SSG and HIT groups, and %change of RPE and PACES **(E)** in the SSG group during the 4-week training period. Week (2–1): the PACES or RPE of the SSG group in week 2 minus that in week 1; SSG: small-sided game; HIT: high-intensity interval training; ^*^significant differences within the HIT group; ^†^significant differences within the SSG group; ^#^significant differences between the SSG and HIT groups; error bars for HR_mean_, %HR_max_, RPE, and PACES is standard deviation (*SD*).

There were no significant interactions (group × time) and main group effects in HR_mean_ (Figure 2A) and %HR_max_ (Figure 2B). A main time effect was found in HR_mean_ (*p* = 0.004; $\eta_{p}^{2}$ = 0.16, minimum) and %HR_max_ (*p* < 0.001; $\eta_{p}^{2}$ = 0.25, minimum), respectively. Although no within-group differences were found in %HR_max_ responses during the 4-week intervention, %HR_max_ in the SSG group were below 90% in week 1 (87.8 ± 2.2%) and week 2 (88.1 ± 2.6%), respectively. %HR_max_ in the HIT group was significantly lower in week 1 than that in week 4 (*MD*: –4.60; 95%*CI*: −8.21 to −0.99; *p* = 0.005; *g* = 1.46, large).

### 3.2. Perceived exertion responses

There were no interactions and main group effects in RPE, but there was a main time effect (*p* < 0.001; $\eta_{p}^{2}$ = 0.31, moderate; Figure 2C). RPE was significantly lower in week 1 than that in week 3 (*MD*: –0.65; 95%*CI*: −1.13 to −0.17; *p* = 0.001; *g* = 1.12, moderate) and week 4 (*MD*: –0.78; 95%*CI*: −1.25 to −0.30; *p* < 0.001; *g* = 1.32, large) in the SSG group, and RPE was lower in week 2 than that in week 4 (*MD*: –0.58; 95%*CI*: −1.05 to −0.10; *p* = 0.008; *g* = 1.25, large). In addition, RPE in the HIT group was lower in week 1 than that in week 3 (*MD*: -0.81; 95%*CI*: −1.23 to −0.40; *p* < 0.001; *g* = 1.77, large) and week 4 (*MD*: -0.79; 95%*CI*: −1.21 to −0.37; *p* < 0.001; *g* = 1.67, large), respectively.

### 3.3. Enjoyment responses

A main group effect was found in PACES (*p* < 0.001; $\eta_{p}^{2}$ = 0.44, moderate), but no interactions and main time effects were noted (Figure 2D). SSG elicited significantly higher PACES than HIT in week 1 (*MD*: 7.70; 95%*CI*: 0.64 to 14.75; *p* = 0.03; *g* = 1.45, large), week 2 (*MD*: 8.44; 95%*CI*: 1.39 to 15.50; *p* = 0.01; *g* = 2.39, very large), week 3 (*MD*: 11.06; 95%*CI*: 4.00 to 18.11; *p* < 0.001; *g* = 1.86, large), and week 4 (*MD*: 7.70; 95%*CI*: 0.64 to 14.75; *p* = 0.03; *g* = 1.56, large), respectively.

Figure 2E presents %change (%Δ) of RPE and PACES of the SSG group in week 2 (2–1), week 3 (3–1), and week 4 (4–1) minus week 1. %change of RPE shows an increasing trend (%Δ_2–1_ = 3.35, %Δ_3–1_ = 9.58, %Δ_4–1_ = 11.53, respectively) and %change of PACES shows a decreasing trend (%Δ_2–1_ = −1.28, %Δ_3–1_ = −0.85, %Δ_4–1_ = −11.49, respectively).

## 4. Discussion

To our knowledge, this is the first study to investigate the acute physiological, perceived exertion and enjoyment responses of female basketball players in 4-week SSG or HIT training programs. Our findings show that SSG was perceived as more enjoyable than HIT as indicated by PACES. SSG and HIT elicited similar physiological and perceived exertion responses, as no significant differences in HR_mean_, %HR_max_ and RPE were found between groups in each week. Although comparisons across time points revealed non-significant differences in HR_mean_ and %HR_max_, SSG elicited HR_max_ response below 90% in the first and second week. Lower HR_max_ responses in the first and second week were accompanied by lower RPE.

Our data show that SSG elicited significantly higher enjoyment responses than HIT in each week of the training intervention. Although there was no data in basketball, our findings are consistent with other research comparing the enjoyment responses between soccer or tennis SSG and HIT (Los Arcos et al., 2015; Kilit and Arslan, 2019; Arslan et al., 2020). From a theoretical point of view, based on the Self-determination theory (Deci and Ryan, 2000), it seemed that SSG (i.e., the presence of ball, teammates and opponents, and the replication of game-like scenarios) result in satisfaction of basic psychological needs—BPN (i.e., autonomy, competence and relatedness) that increased intrinsic motivation and enjoyment response. In brief, feeling of autonomy and relatedness could be easily satisfied in SSG where the players had a freedom to regulate their own actions (e.g., dribbling, shooting) and feel affiliated and connect to the team, respectively. Likewise, in 2 vs. 2 scenario players were able to showcase their basketball ability more frequently than in a 5 vs. 5 scenario, in which they might not have as many chances to score or be creative, which could satisfied their psychological need for competence. It can be speculated that SSG provided stimulating, learning and challenging environment that facilitated satisfaction of BPN, which in turn could increase intrinsic (i.e., autonomous) motivation and therefore positively affect enjoyment response (Selmi et al., 2020) and perceived effort (Monteiro et al., 2018). Moreover, it is reasonable to believe that athletes recognized SSG as meaningful activities and training environment that provided opportunities for learning new sport-specific skills which could induce long-lasting motivation (Dismore and Bailey, 2011). Therefore, SSG seems a more effective training method than HIT (i.e., simply running at predetermined intensity) for increasing exercise motivation and adherence. We recommend that coaches schedule SSG training more frequently than HIT for players who regularly participate in training and competitions in order to maintain players’ exercise enthusiasm.

Similar physiological and perceived exertion responses in SSG and HIT observed in our study indicates that the two training approaches could elicit similar physiological training stimuli. Despite the fact that there are fewer studies on female basketball players (Reina et al., 2020), making it difficult to compare players of similar levels, our findings are consistent with other research investigating male basketball players (Delextrat and Martinez, 2014; Delextrat et al., 2018) and male soccer players (Dellal et al., 2012). In contrast, some studies indicated that SSG could elicit lower perceived exertion compared with HIT since it is more enjoyable (Kilit and Arslan, 2019; Arslan et al., 2020). The similar perceived exertion between the two training approaches in our study could be explained by the fact that the SSG format (2 vs. 2) results in higher training intensity than formats including larger team sizes (Castagna et al., 2011; Klusemann et al., 2012).

SSG elicited %HR_max_ responses below 90% in first and second week. Given the notion that training at high HR zones (above 90% of HR_max_) is considered to be more effective than lower HR zones (Delextrat and Kraiem, 2013; Malone et al., 2019), cardiovascular stimuli in the first 2 weeks were probably insufficient and needed to be optimized. Significant lower RPE in first and second week (7.2–7.4) compared with that in third and fourth week (7.8–8.0) also support the inference. Given that the main purpose of pre-season training is to develop sport-specific performance and maximize training effectiveness (Paul et al., 2019), it is practical to appropriately increase the training stimulus during the SSG intervention program. Researchers suggested that SSG with longer duration bouts could elicit greater physiological and perceived exertion responses (Klusemann et al., 2012; Conte et al., 2016). Given that SSG elicited above 90% of HR_max_ responses and 7.8–8.0 of RPE in third and fourth weeks, the duration of each bout of SSG in first and second weeks could be increased to achieve larger adaptations. Therefore, we infer that it would be appropriate to set the SSG duration at least 7.5 min in one training session during first week of the intervention, and gradually increase training durations in the subsequent weeks. Similar patterns could be used by researchers and practitioners to determine whether implemented training programs achieve optimal training stimuli, with the goal of optimizing intervention effectiveness.

Manipulating team sizes, court sizes, and rules could impact physiological and perceived exertion responses during SSG training (Clemente, 2016; O'Grady et al., 2020). In our study, 2 vs. 2 SSG was performed given that smaller team sizes allow for a larger relative playing area per player and greater freedom of movement, resulting in greater physiological and perceived exertion responses (Castagna et al., 2011). Furthermore, we used half-court (14 × 15 m) playing area to allow more technical actions performed (Klusemann et al., 2012; Atli et al., 2013) and more players simultaneously involved (up to 8 players performing 2 vs. 2 drills at the same time). Although full-court SSG are more likely to elicit greater training stimuli because of conducting rapidly transitions up and down the court (Atli et al., 2013), researchers showed no differences in HR responses between full-court and half-court SSG (Klusemann et al., 2012; Bredt et al., 2020). In addition, we prescribed that no times-outs or free-throws were rewarded (Delextrat and Kraiem, 2013), the ball was replaced immediately when out of play (Conte et al., 2016), and a 12-s shot clock was used (Klusemann et al., 2012) to avoid interruptions and enhance the exercise intensity. We also arranged the regular offensive and defensive schemes (e.g., man-to-man defensive) to standardize technical–tactical parameters. As a result, half-court, 2 vs. 2 SSG combined with modified rules seems to preserve the relative consistency of SSG’s content and assist players in receiving optimal cardiovascular stimuli while maintaining relatively high enjoyment during training.

From a practical perspective, coaches prefer to increase the frequency of SSG in training programs due to the desired cardiovascular stimulus and high enjoyment responses elicited during training. However, our findings show that gradually increased RPE was accompanied with decreased PACES during the 4-week progressive SSG intervention (Figure 2E). Research (Fernandez-Rio et al., 2014) also suggested that performing high-intensity training sessions for 3 weeks would lower self-determined motivation, which is mainly influenced by perceived enjoyment responses during training. Accordingly, we infer that progressive SSG interventions appear to slightly reduce enjoyment responses and motivation due to increased training intensities and loads. Furthermore, the high physiological and perceived exertion responses in SSG, combined with its frequent use, are likely to cause insufficient recovery and raise the potential risk of overtraining or injury (Clemente, 2016). Thus, monitoring acute physiological, perceived exertion and enjoyment responses during each training session is necessary to avoid overuse of SSG and is beneficial in determining its optimal dose.

## 5. Limitations

There are several limitations of the study that should be acknowledged. First, this study did not assess blood parameters (e.g., blood lactic acid) which could help in additional explanation of the underlying mechanisms of the physiological load. Second, the PACES was filled out anonymously, so the correlation between PACES and RPE could not be calculated. Third, the study was performed with sub-elite female basketball players and included a relatively small sample size, so extrapolation of the findings to elite and male players should be taken with caution. Forth, motivational factors were not measured which thwart establishing relationship between players´ motivation, satisfaction of basic psychological needs and enjoyment and perceived effort response in SSG and HIT. Finally, the study did not incorporate measurements of players´ external load during SSG, which could help to additionally explain the obtained results.

## 6. Conclusions and practical application

Overall, SSG and HIT elicit similar physiological and perceived exertion responses during training sessions, but SSG is more enjoyable and therefore it is more likely to increase exercise motivation and adherence comparing to HIT. The current study encourages practitioners and researchers to incorporate SSG training programs during pre-season for collegiate female basketball players. It seems that half-court, 2 vs. 2 SSG training format with modified rules and lasting ≥ 7.5 min should be prescribed as an enjoyable training alternative to provide optimal cardiovascular stimuli (> 90% of HR_max_) for female basketball players. Coaches should frequently ask basketball players to set the playing rules by themselves during SSGs, which in turn could increase their autonomous (i.e., intrinsic) motivation and therefore positively affect enjoyment response and perceived effort. Moreover, we recommend coaches to use SSGs as training environment that provides opportunities for learning and improvement of specific skills (e.g., dribbling and/or scoring only with a non-dominant hand) and implementation of new tactical ideas and concepts both in offense (e.g., scoring only after pick and roll play) and defense (e.g., switching or setting a trap). In this way, players may recognize the training setting as constructive, beneficial and meaningful, which could induce long-term motivation and improvement.

## Data availability statement

The raw data supporting the conclusions of this article will be made available by the authors, without undue reservation.

## Ethics statement

The studies involving human participants were reviewed and approved by the Regional Ethical Review Board in Hangzhou (IR00350-SPT-2020). The patients/participants provided their written informed consent to participate in this study.

## Author contributions

JZ, FX, and JX: conception and design. JZ and FX: analysis and interpretation of the data. JZ, HP, and FX: drafting the article and revising it critically for important intellectual content. All authors contributed to the article and approved the submitted version.

## Conflict of interest

The authors declare that the research was conducted in the absence of any commercial or financial relationships that could be construed as a potential conflict of interest.

## Publisher’s note

All claims expressed in this article are solely those of the authors and do not necessarily represent those of their affiliated organizations, or those of the publisher, the editors and the reviewers. Any product that may be evaluated in this article, or claim that may be made by its manufacturer, is not guaranteed or endorsed by the publisher.

## Footnotes

^1^ http://www.jasp-stats.org
